# The effect of graphite and carbon black ratios on conductive ink performance

**DOI:** 10.1007/s10853-017-1114-6

**Published:** 2017-04-27

**Authors:** Chris Phillips, Awadh Al-Ahmadi, Sarah-Jane Potts, Tim Claypole, Davide Deganello

**Affiliations:** Welsh Centre for Printing and Coating, College of Engineering, Swansea University, Bay Campus, Crymlyn Burrows, Swansea, SA1 8EN UK

## Abstract

Conductive inks based on graphite and carbon black are used in a host of applications including energy storage, energy harvesting, electrochemical sensors and printed heaters. This requires accurate control of electrical properties tailored to the application; ink formulation is a fundamental element of this. Data on how formulation relates to properties have tended to apply to only single types of conductor at any time, with data on mixed types of carbon only empirical thus far. Therefore, screen printable carbon inks with differing graphite, carbon black and vinyl polymer content were formulated and printed to establish the effect on rheology, deposition and conductivity. The study found that at a higher total carbon loading ink of 29.4% by mass, optimal conductivity (0.029 Ω cm) was achieved at a graphite to carbon black ratio of 2.6 to 1. For a lower total carbon loading (21.7 mass %), this ratio was reduced to 1.8 to 1. Formulation affected viscosity and hence ink transfer and also surface roughness due to retention of features from the screen printing mesh and the inherent roughness of the carbon components, as well as the ability of features to be reproduced consistently.

## Introduction

Conductive carbon materials, in particular those derived from graphite and carbon black, have been used in the manufacture of inks and coatings for a range of printed electronic applications including batteries [1] and supercapacitors [2, 3], electrochemical sensors [4], PCB resistors [5] printed heaters [6] and more recently in solar energy harvesting [7]. Carbon inks have a host of favourable characteristics that allow them to be used in these applications, including chemical inertness, the ability to be modified or functionalised in the case of electrochemical sensors and ability to act as intercalating materials in the case of energy storage, as well as low cost and disposability. Graphite is a layered planar structure, typically tens of microns in length, and is conductive primarily along its planes. Carbon black on the other hand is a sub-micron scale high surface area particle with a roughly spherical shape [8]. Within a polymeric bonder, the complimentary interaction between these two types of carbon gives rise to a conductive matrix which is substantially more conductive than if these materials are used in isolation. The small carbon black particles readily disperse and form conducting bridges between the graphite rich areas of the composite matrix [9]. Carbon nanotubes have been studied extensively of late as alternative filler materials [10], but they are limited by their very high cost and tendency to agglomerate, making them difficult to process for volume applications [11].

Throughout the printed electronics industry, the dominant deposition method is screen printing due to its relative simplicity, low cost, versatility and maturity in the sector [12]. The screen printing process has the ability to deposit thick layers of viscous slurries be they conductive metals or carbons, or indeed insulators and dielectrics, with high functional material loadings, and hence performance for the various applications. Carbon inks for screen printing are a commercially established technology, particularly in applications such as blood glucose sensors and while there is some literature on the influence of conductive carbon ratios in paints and composites [9, 13, 14], there is a lack of published literature into the interaction between graphite and carbon black and the effect of the ratios of these ingredients in inks. An abundance of composites studies uses the classical percolation theory to model electrical properties alongside experimental testing, but these only consider single types of carbon in any system [15, 16]. Control of the conductivity of screen printed features is vital for achieving the correct performance and consistency in printed devices and is governed by the inherent conductivity of the deposited material as well as the topography of the printed features. The electrical properties of such inks that use different carbon types are due to a complex series of interactions between the morphology and size of the individual components, their inherent properties and the processing methods used to disperse them. Therefore, in order to improve the understanding of how formulation of the ink affects the printed product, a study was undertaken in which a series of inks were manufactured under consistent and controlled conditions using different ratios of graphite, carbon black and polymer. The effect on ink viscosity, screen printing deposition in terms of thickness and surface roughness, and crucially conductivity was then established.

## Materials and methods

### Ink formulation

Graphite (*Timrex* ^*®*^ *SFG15*, Imerys Graphite and Carbon—typical D90 17.9 µm according to manufacturer) and carbon black (*Conductex SC Ultra*, Birla Carbon—mean particle size 20 nm according to manufacturer) were used to produce a total of ten batches of screen printing carbon ink with different graphite to carbon black ratios of 0.5, 1, 1.8, 2.6 and 3.2 to 1. Each of these ratios was produced at two different total carbon concentrations; a higher carbon concentration of 29.4% by mass and a lower carbon concentration of 21.7% by mass (with the remaining content being 70.6 and 78.3% resin dispersion, respectively). Five higher total carbon concentration inks were made first by adding carbon black and graphite to a pre-made vinyl resin base (with 15% by weight dry polymer, VINNOL (Wacker Chemie AG) in 4-hydroxy-4-methylpentan-2-one) to produce a total mass of 200 g as listed in Table 1. The total carbon content was kept the same, but each formulation used different graphite to carbon black ratios. Carbon materials were added gradually and mixed by hand, with carbon black added before the graphite. The ink slurries were allowed to wet overnight before milling. Milling was carried out using an EXAKT80E three roll mill (EXAKT Advanced Technologies GmbH) with the same processing conditions used for each ink as shown in Table 2. To produce the inks with lower total carbon (i.e. higher resin concentration), but identical ratios of graphite to carbon black, samples of each of the inks were taken and 26.1 g of resin was added to 73.9 g of each ink and mixed by hand. An additional milling cycle, using the same settings as passes 3 and 4, was used to ensure good mixing. For each type of ink, a single batch was used for printing and analysis.

**Table 1:** Ink batch composition for parent higher carbon content inks

Graphite/carbon black ratio	0.5	1	1.8	2.6	3.2
Graphite %	9.8	14.7	18.9	21.3	22.4
Carbon black %	19.6	14.7	10.5	8.1	7.0
Resin base %	70.6 (of which 15% solid, 85% solvent)

**Table 2:** Three roll mill settings for carbon ink manufacture

Pass #	Back gap (micron)	Front gap (micron)	Speed (front roller) rpm
1	60	15	200
2	40	10	200
3	20	5	200
4	20	5	200

### Analysis

#### Viscosity measurement

The viscosity of the ink formulations was measured using a rheometer (Gemini Bohlin Nano, Malvern Instruments) with a 2° 20 mm stainless steel cone and a parallel plate held at 25 °C over a shear rate range of 1 to 200 s^−1^. Ink viscosity was measured as the shear rate was increased to 200 s^−1^ and then reduced back to 1 s^−1^. Three measurements were taken for each ink and the results averaged.

#### Printing methodology

Printing was carried out on a DEK 248 flatbed screen printing machine using a polyester mesh with 61 threads per cm, 64 µm thread diameter and 13 micron emulsion, 2 mm snap-off, 65–70 *Shore A* hardness diamond squeegee of 130 mm length with a 12-kg squeegee force and print/flood speeds of 70 mm s^−1^. The substrate was PET (polyethylene terephthalate—Melinex^®^ 339, DuPont Teijin Films (330 µm thickness) opaque white. The print image included a series of 25-mm-long lines of differing widths and a 45-mm square solid patch for sheet resistance assessment. Printed samples were dried in a box oven at 100 °C for 30 min and left overnight before measurement.

#### Printed ink geometry and surface topography measurement

White light interferometry (NT2000, Veeco Instruments, Inc., Plainview, NY, USA) was used to measure a full three-dimensional surface profile over the edge of the solid print so that the printed ink film thickness could be evaluated. Five times magnification was used, giving a measurement area of 1.2 mm by 0.93 mm (at a resolution of 736 × 480 pixels with sampling at 1.67-µm intervals). The ink film thickness was calculated as the average height of the substrate subtracted from the average height of the ink, excluding the print edges where there tended to be a lip or a decline in ink film thickness depending on the print orientation. A total of 12 measurements were taken for each ink type (4 measurements each on 3 print samples) (prints 3, 4 and 5 at the centre of each edge of the printed square). Average surface roughness measurements (*Sa*) over the printed area were also taken away from the edges. To provide a visual representation of the surfaces, 3D microscopy (Alicona Infinite Focus G5 microscope (Alicona Imaging GmbH)) was used for its ability to more effectively capture the surface form of carbon ink, which was resolved in less detail for white light interferometry. Finally, in order to visualise particle distribution within the dried ink layers, scanning electron microscope images were taken using a JEOL JSL-7800F SEM.

Line geometry was assessed using white light interferometry at 5× magnification. 300 and 500 µm nominal width lines were measured for print number 3 at three points along the line. Geometric features were evaluated by taking discrete measurements over the 1.2-mm-length sections measured by the interferometer (736 measurements at 1.67-µm intervals for each measured section). From this the standard deviation in line width and thickness was calculated.

#### Resistance measurement

Sheet resistance of the 45-mm square was measured using the four-point method. A SDKR-25 probe (NAGY Messsysteme GmbH) with a tip distance of 2.5 mm was used with a Keithley 2000 digital multimeter. Taking into account the dimensions of the samples, a correction factor of 4.3 was used as proposed by Smits [17]. Line resistance was measured using the same multimeter in two-point mode. Sheet resistances are shown as measured and, to better indicate the relative performance of the inks, resistivities were calculated as the product of sheet resistance and ink film thickness. A total of 20 measurements were taken for each ink type (5 measurements each on print samples 3, 4, 5 and 6). For printed lines, two-point measurement was used with the multimeter.

## Results

### Ink viscosity

**Figure 1:**
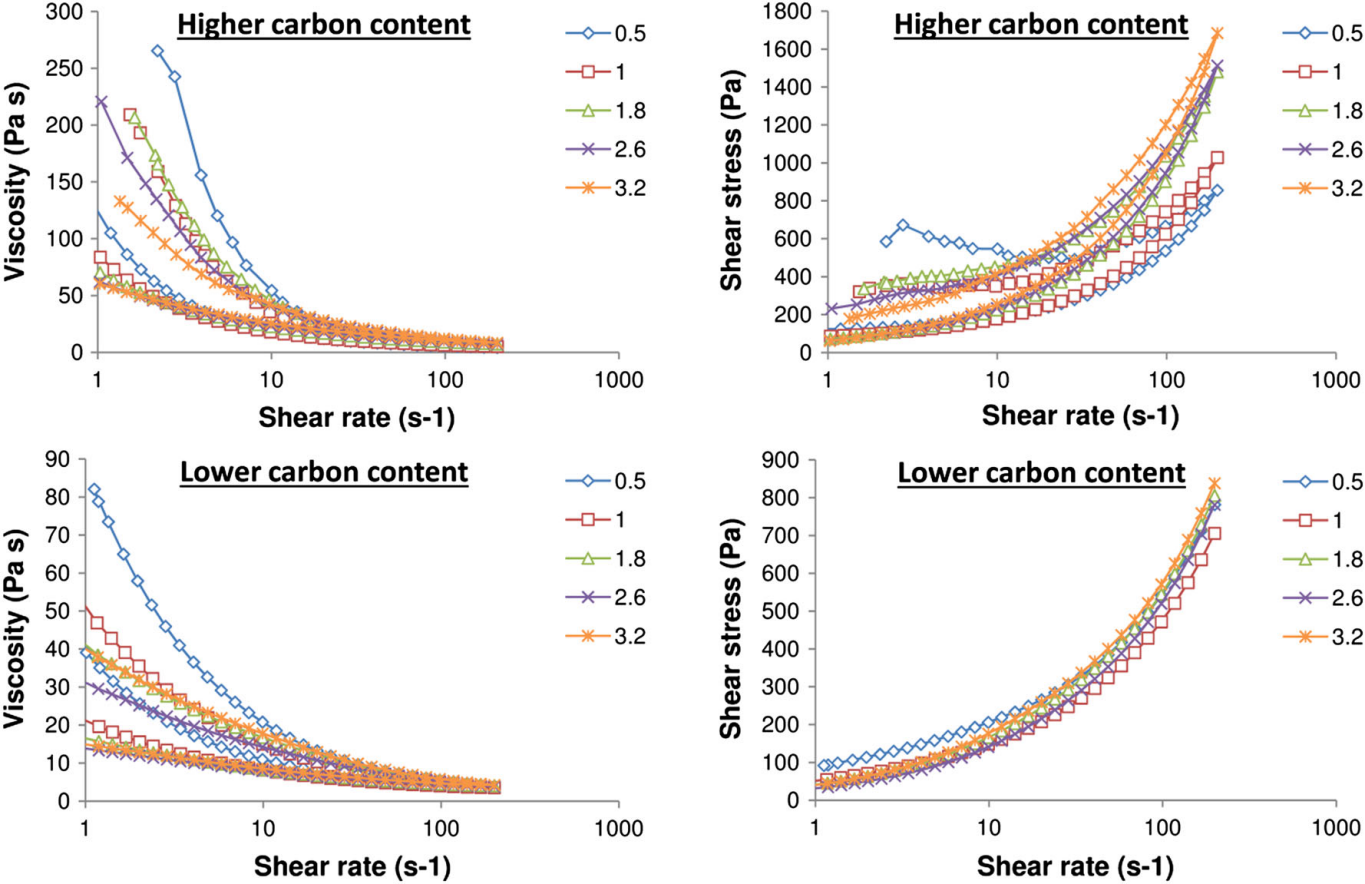
Viscosity (*left*) and shear stress (*right*) of inks with varying graphite to carbon black ratios as a function of shear rate

The viscosity and shear stress levels of the inks as they were sheared up to a shear rate of 200 s^−1^ and relaxed back to 1 s^−1^ are shown for all inks in Fig. 1. Shear stress levels are also shown for the increasing shear phase only. All inks demonstrated shear thinning behaviour, with viscosity decreasing as shear rate was increased. The higher carbon content inks had higher viscosity, and there was a general trend of higher carbon black content inks having higher low shear viscosities and shear stress. Carbon black has a much smaller particle size and higher specific surface area than graphite. Higher proportions of carbon black in the ink would therefore result in a greater number of particle–particle interactions, as well as smaller inter-particle spacing, resulting in more opposition to inter-particle slippage, and thus higher viscosity at low shear rates [18]. Consequently, as more of the carbon black was substituted for graphite, this viscosity fell. However, it should be noted that as shear rate was increased, the higher carbon black inks tended to shear thin more readily, resulting in lower viscosities at the high shear rate range. This might be due to these relatively weak particle–particle interactions being broken down under high shear. All inks showed only partial recovery of viscosity on reduction of the shear rate.

### Ink film thickness and surface form

#### Solid patch

**Figure 2:**
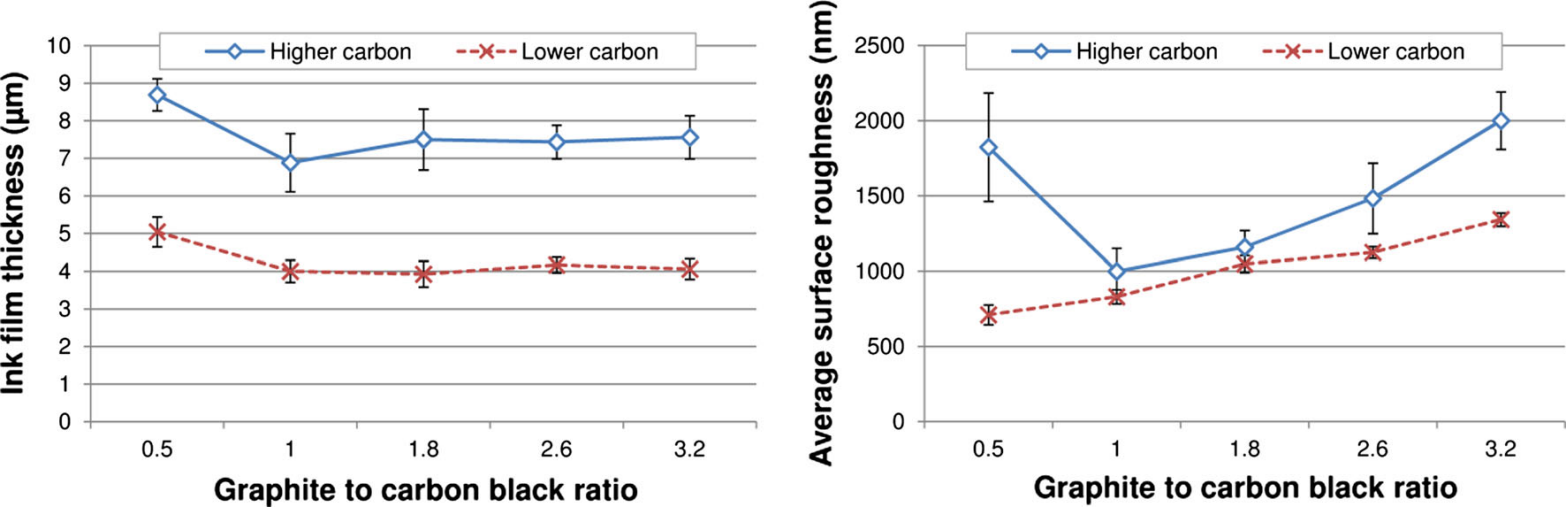
Ink film thickness (*left*) and average surface roughness (*right*) of solid prints using the various inks (*error bars* show standard deviation)

The ink film thickness and surface roughness produced from the various ink batches are shown in Fig. 2. For both carbon concentrations, the lowest graphite content inks appeared to give significantly thicker ink film thicknesses than the other inks, which were broadly similar to one another at a given resin concentration. The lower carbon content inks were deposited in substantially thinner films than their equivalents with higher carbon content. Part of this can be attributed to the greater shrinkage during drying resulting from the lower solid content (average solid content was 39.5% for higher carbon content inks and 33.7% for lower carbon content inks), but the magnitude of the difference also suggests a substantially thinner wet deposit in the lower carbon content inks.

**Figure 3:**
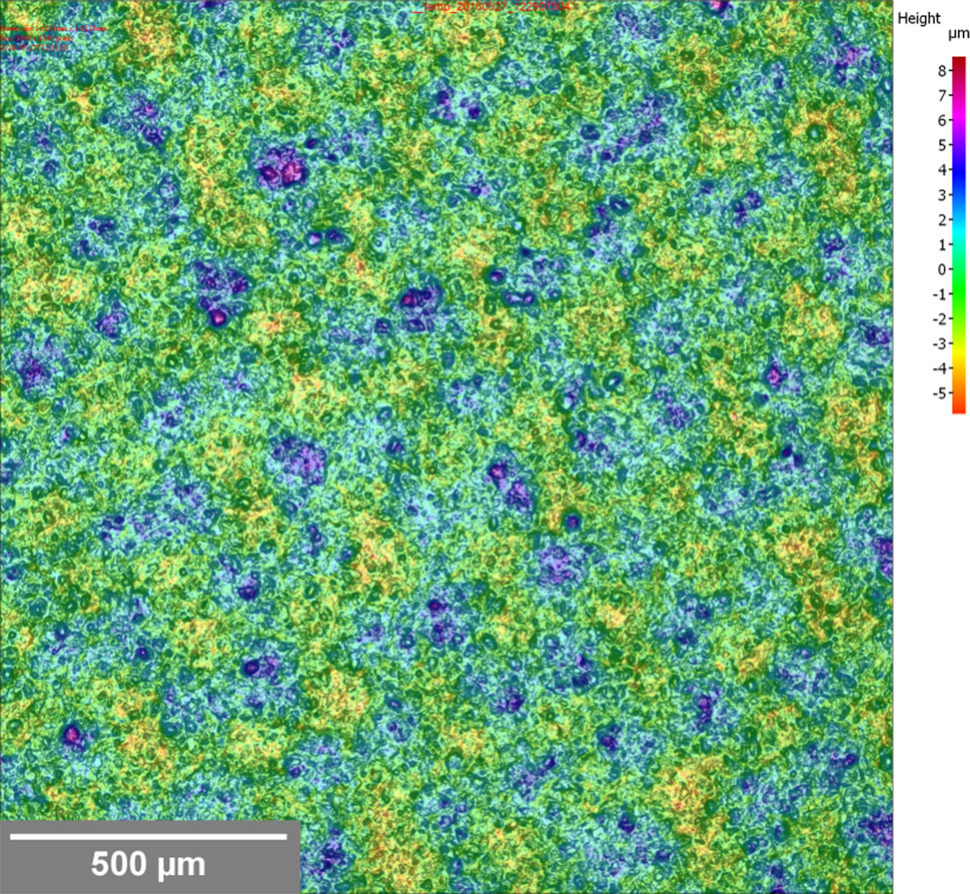
Visible light microscope images for 0.5 to 1 graphite to carbon black ink at higher carbon content, with *overlaid colour* contour indicating local surface height and patterning on print surface due to “mesh marking”

**Figure 4:**
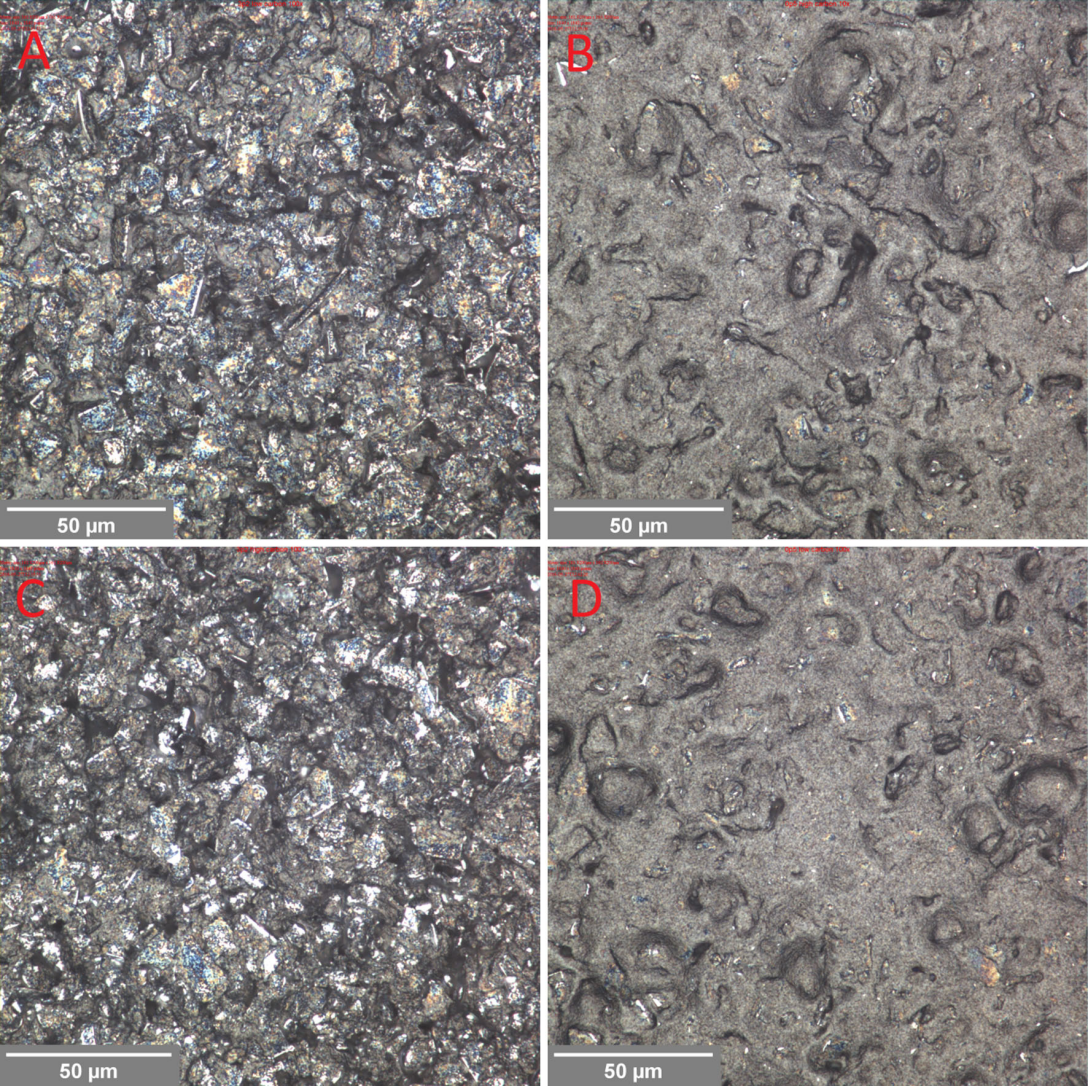
Visible light microscope images showing surfaces of* lowest* and* highest* graphite content inks: 3.2 parts graphite to carbon black (**a**) and 0.5 parts graphite to carbon black (**b**) at high carbon content, 3.2 parts graphite to carbon black (**c**) and 0.5 parts graphite to carbon black (**d**) at low carbon content

The two factors which contribute to roughness are the underlying topography of the print and the roughness due to the particles. The so-called mesh marking, regular features corresponding with the frequency of the mesh (61 threads per cm), is more evident at high viscosity, while roughness due to particles becomes more evident as the graphite to carbon black ratio is increased. The higher carbon content inks had substantially higher surface roughness than their low carbon equivalents, displaying regular surface features due to the printing mesh. This marking was most evident in the 0.5 graphite to carbon black at high carbon concentration (Fig. 3) and resulted in a high surface roughness value. With the exception of this particular ink, both sets of ink became progressively rougher as the graphite content was increased. This was due to the larger particle size of the graphite component of the ink having a progressively more dominant effect on the surface. The ink film thickness and roughness values were substantially more consistent for the lower carbon content inks. This suggests a more even surface due to greater slumping of the ink after the mesh is released from the substrate during snap-off. The 0.5 graphite to carbon black ratio inks were substantially different in rheology from the other inks formulations, with much higher low shear viscosities and steeper shear profiles. These inks also gave higher ink film thicknesses than the other inks, suggesting a link between viscosity and ink film thickness, but also roughness due to replication of features due to the screen due to an inability of the ink to readily slump. At the start of a printing cycle, ink is in a low shear rate rest condition where it is at high viscosity. The ink is then pushed by the squeegee over the screen surface, at an intermediate shear rate before being forced through the mesh at a high shear rate at which point the ink is at its lowest viscosity. The shear rate then reduces and the ink recovers its structure and hence viscosity. This recovery will determine the degree of slumping in the print [19]. The surfaces of the prints with lowest and highest graphite content are compared optically in Fig. 4. The images demonstrate the difference in surface texture and the presence of greater numbers of large particles in the highest graphite concentration inks.

**Figure 5:**
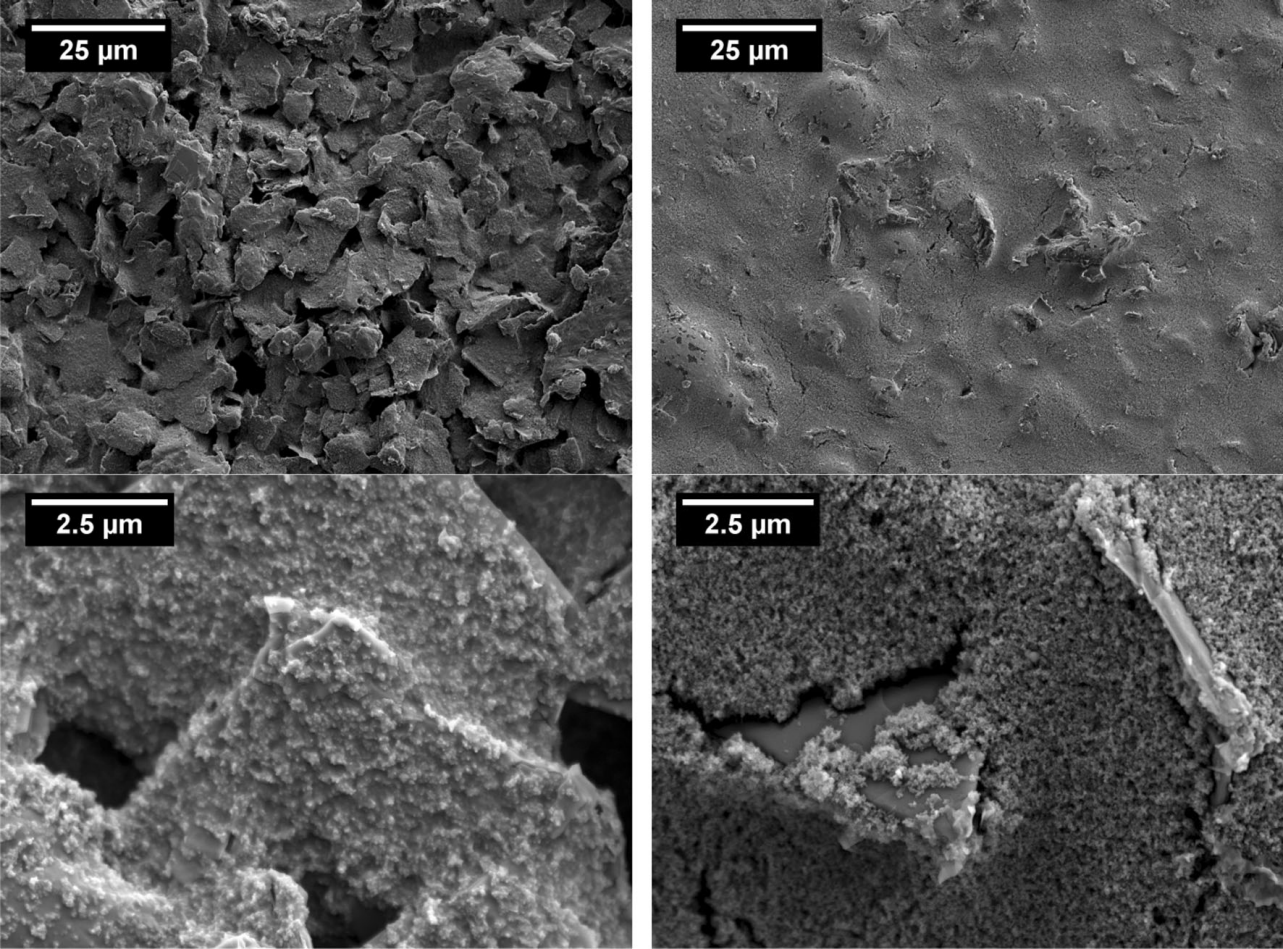
Scanning electron microscope images showing surfaces of* lowest* and* highest* graphite content inks: 3.2 parts graphite to carbon black (*left*) and 0.5 parts graphite to carbon black (*right*) at high carbon content at ×1000 (*top*) and ×10000 (*bottom*) magnifications

Scanning electron microscope images are shown in Fig. 5 for prints made using 3.2 parts graphite to carbon black and 0.5 parts graphite to carbon black, both at the higher carbon content. At the higher graphite concentration, the graphite particles were close together and coated in a polymer/carbon black matrix which also acted as conductive filler bridging between neighbouring graphite particles. At low graphite content, the graphite particles tend to be isolated from one another, with the polymer/carbon black matrix forming the bulk of the ink and heavily coating most of the graphite particles.

#### Lines

**Figure 6:**
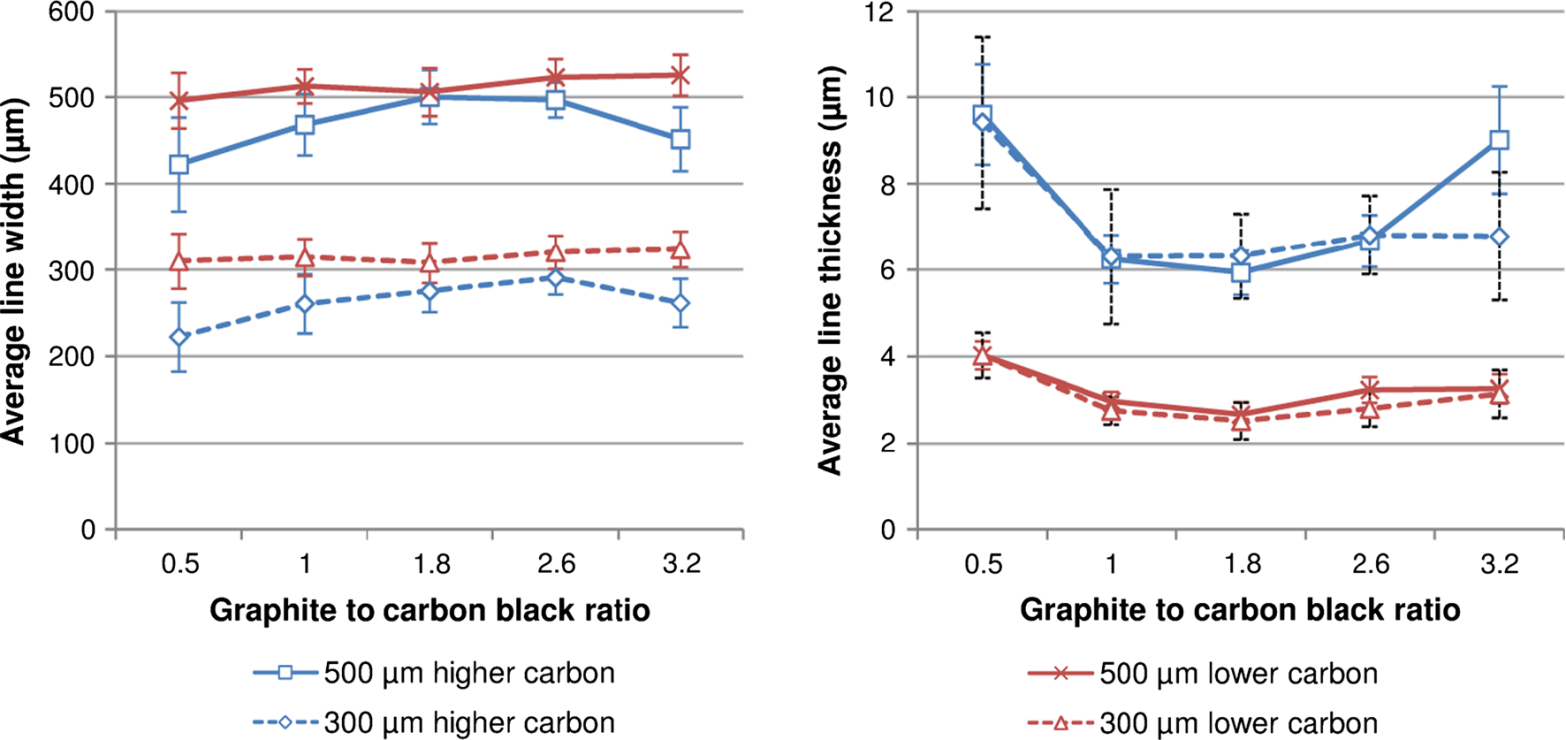
Line width (*left*) and ink thickness (*right*) obtained using the various inks (*error bars* show standard deviation within the measured lines)

**Figure 7:**
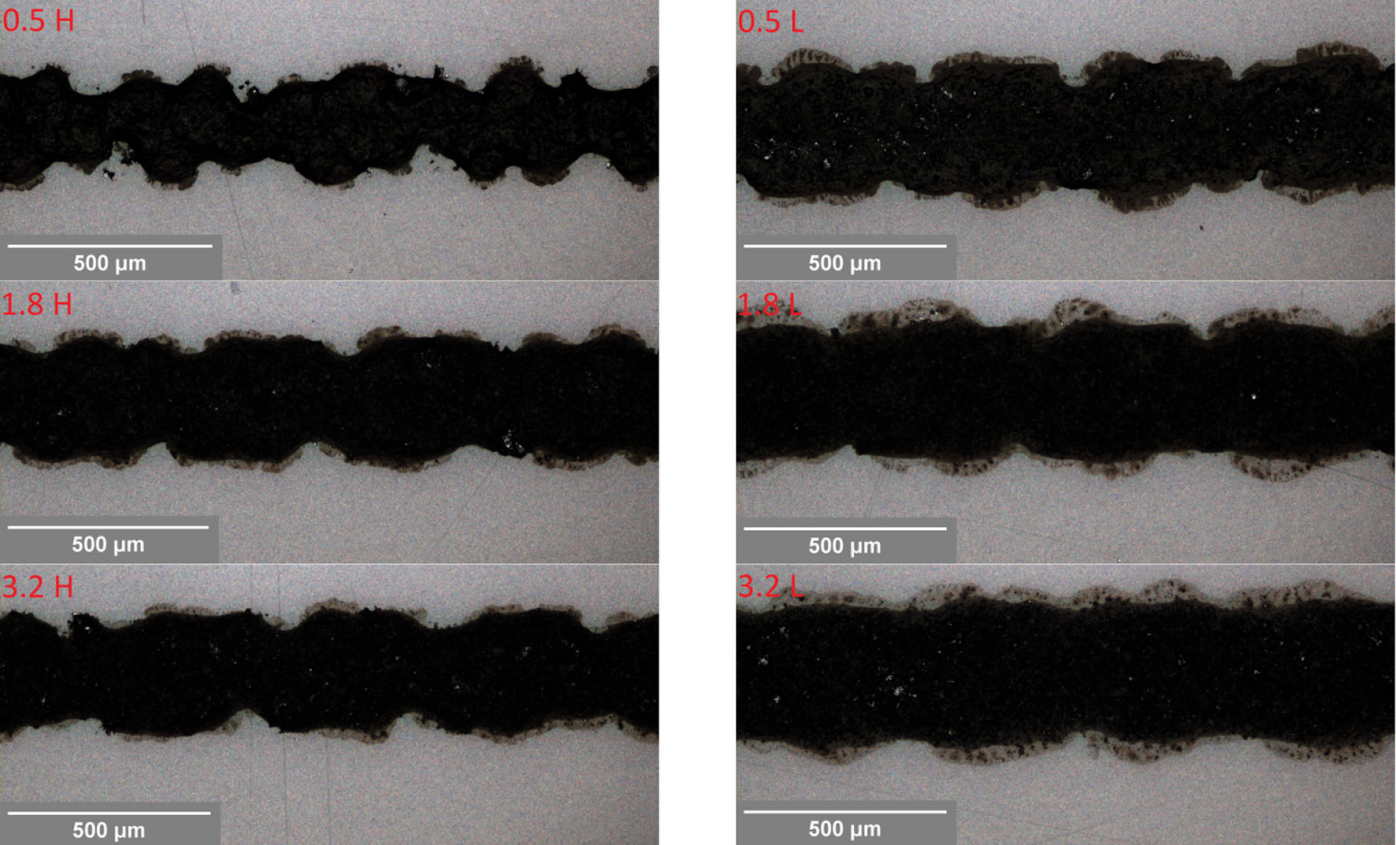
Visible light microscope images showing variations in line quality for 300 micron nominal width lines: 0.5 to 1 graphite to carbon black at high (0.5 H) and low (0.5 L) carbon content, 1.8 to 1 at high (1.8 H) and low (1.8 L) carbon content, 3.2 to 1 at high (3.2 H) and low (3.2 L) carbon content

Line width and ink thickness obtained using the various inks are shown in Fig. 6. Lines printed using the higher carbon content ink were thicker and narrower than the lines produced using the lower carbon inks. The lower viscosities of the lower carbon inks allow the printed lines to slump more readily. The narrowest and thickest lines were produced using the most viscous ink which was the high carbon with 0.5 parts graphite to carbon black. Previous studies [20] have demonstrated decreasing line width and increasing printed line thickness as solid content and hence viscosity of a carbon ink was increased. Higher carbon inks were also less consistent in terms of their line width and thickness over the length of the line than the low carbon ink lines. As in the solid print, the lines from higher carbon inks were also prone to mesh effects with a saw-tooth edge and uneven profile particularly evident on the higher carbon with 0.5 parts graphite to 1 carbon black. This was reduced by both reducing total carbon content and increasing the ratio of graphite to carbon black. This is highlighted in Fig. 7 which compares a 300 micron nominal width lines from a range of inks.

### Electrical properties

#### Sheet resistance

**Figure 8:**
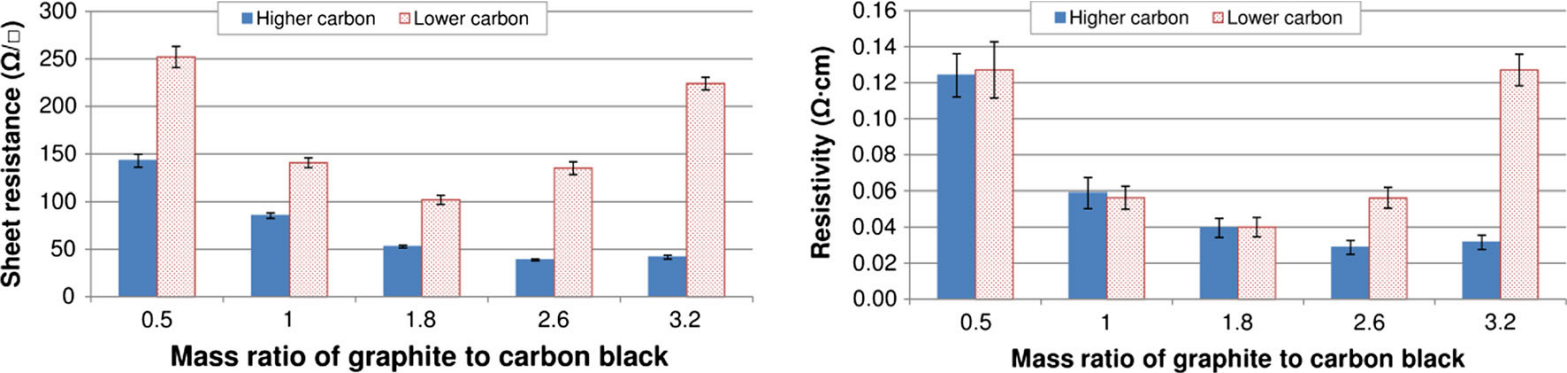
Sheet resistance (*left*) and resistivity (*right*) for the various inks (*error bars* show standard deviation)

Sheet resistance and resistivity are compared for the different inks in Fig. 8. The higher and lower carbon content inks gave optimum conductivities at different graphite to carbon black ratios. For the higher carbon content inks, a graphite to carbon black ratio of 2.6 to 1 gave the most conductive ink with a resistivity of 0.029 Ω cm. An increase in graphite content to 3.2 to 1 gave a small increase in resistivity. For the lower carbon content inks, the measured resistances were higher. This is due in part to the lower thicknesses at which the inks are printed (Fig. 2). The lowest resistance for these inks was achieved with a reduced graphite to carbon black ratio of 1.8 to 1, with a resistivity of 0.040 Ω cm. Increases in graphite content beyond the optimum loading gave rise to a much more significant increase in sheet resistance than that observed for the higher carbon content inks. A possible explanation for this is that as the carbon black content is reduced, the polymer/carbon black matrix between the graphite particles has a lower carbon black concentration which makes it a relatively poor conductor. Carbon black to polymer ratios were 33% lower in the lower carbon inks. The decline in performance with respect to the higher carbon inks was also exacerbated by the greater distance between graphite particles as polymer loading was increased.

The sheet resistance values are due to both differences in the inherent resistivity of the ink and the thickness at which it is deposited. When comparing the prints in terms of resistivities, the performance of both sets of inks was broadly similar at 0.5, 1 and 1.8 graphite to carbon black ratios. As this ratio was increased, the lower carbon content ink became substantially less conductive by comparison. Given the higher polymer content, a higher resistivity would be expected across the full range; part of this may be due to the difficulty in obtaining a representative ink film thickness measurement for the inks (note that the standard deviation takes into account variation in both sheet resistance and thickness measurement). However, the roughness and consistency of the deposit also need to be taken into account. In this case, the increased resin content in the lower carbon content inks resulted in smoother, more consistent printed films without the mesh marking seen at the higher viscosity high carbon content inks (Fig. 3).

#### Resistance of printed lines

**Figure 9:**
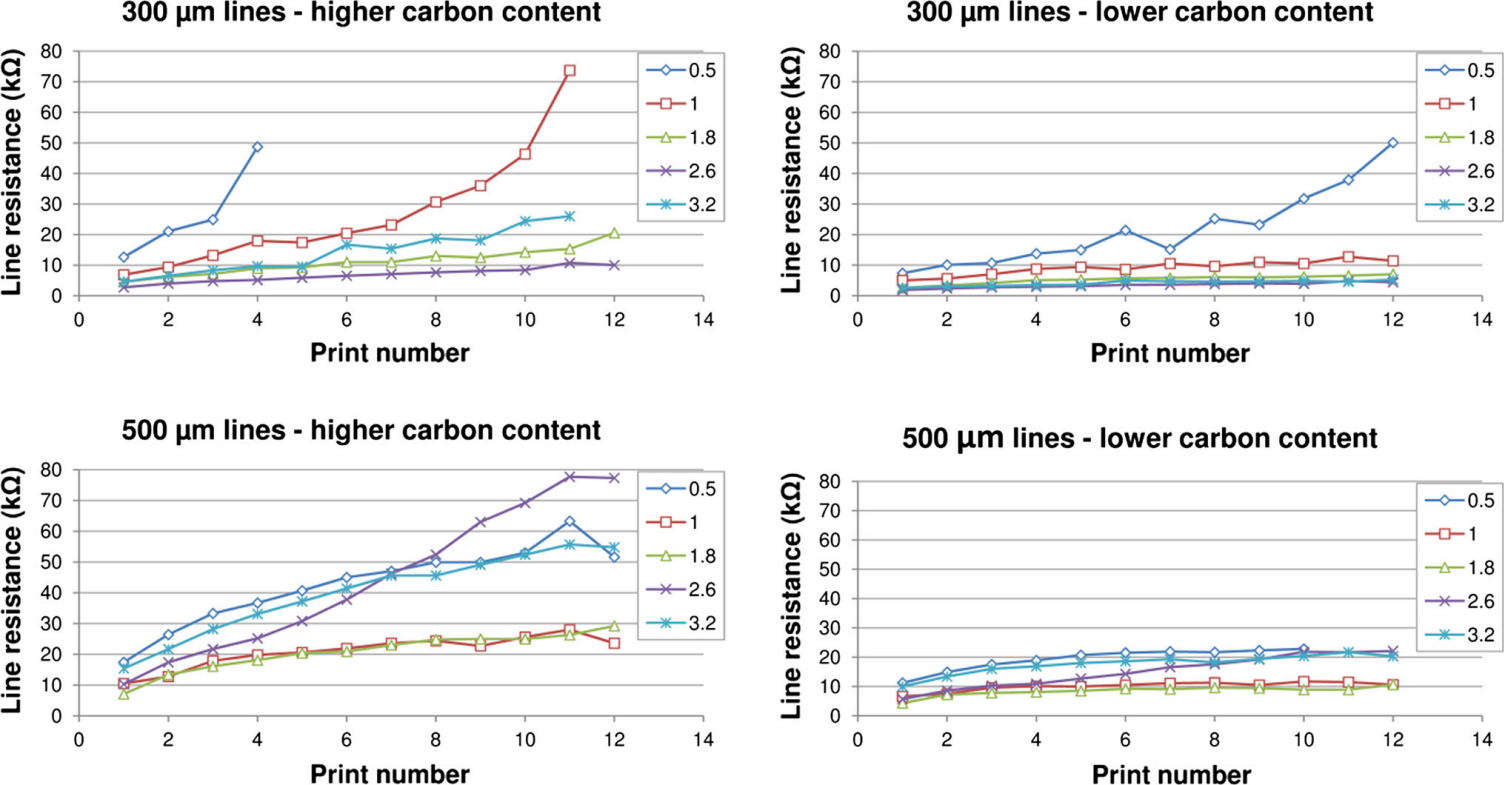
Variation in line resistance over the print run for 25-mm-*long lines* for inks using 0.5, 1, 1.8, 2.6 and 3.2 mass ratios of graphite to carbon* black* at high (*left*) and low (*right*) carbon contents

The resistance of 300 and 500 micron nominal width lines of 25 mm in length is shown in Fig. 9. In general, the lines produced from higher carbon content inks gave lower resistance than those produced using lower carbon content inks due to the inherently higher conductivity of the inks and greater thickness of the deposit. However, as well as the inherent conductivity of the printed ink, the performance of a printed line is dictated by its geometry. For the higher carbon content inks, some of the finer lines were not rendered effectively, and as the print run progressed there was a tendency for the ink to dry in and progressively block small features in the screen. The lines did not remain continuous over their length and therefore were no longer conductive. Lines became less viable at low line width, high print run numbers and high carbon black ratios. However, for lower carbon content inks, all lines were produced throughout the print run, and some lines showed lower resistances than their counterparts with higher carbon content, despite having lower conductive content and ink film thickness. This suggests that the higher resin content inks were better able to produce consistent fine lines and print for longer without drying in.

Poor line reproduction for the higher carbon black content inks (as illustrated in Fig. 6) gave a higher resistance than would be expected based on the relative resistivities as indicated by the sheet resistance data as well as the amount of ink deposited. As the viscosity of the ink was reduced, the inks were better able to slump and form an even line.

## Discussion

Maximum conductivity was obtained when using a 29.4 mass per cent carbon ink with a ratio of graphite to carbon black of 2.6 to 1. However, inks made with lower carbon content (21.7%) required a lower graphite to carbon black ratio (1.8 to 1) to optimise conductivity. The range of sheet resistances obtained in the testing was 38.7–252.2 Ω/□ with a corresponding resistivity range of 0.029–0.127 Ω cm. Further increases in total carbon might improve conductivity but at the expense of ink adhesion, durability and ease of processing. Increases in screen emulsion and thread diameter will produce thicker printed films with lower resistance. However, to obtain lower conductivities, alternative less conductive filler materials would be required. This could not be achieved by reduction in carbon content alone as this would reduce viscosity and affect both ink transfer and print quality. Using graphite as the only carbon material does give a highly resistive ink, but the material is very difficult to process.

Higher carbon black content gave higher rest viscosity, greater shear thinning and a lower high shear viscosity. There appeared to be an association between the viscosity at low shear rates and ink film thickness, with the stiffer inks giving thicker printed ink films and retaining surface features due to the mesh. Inks with lower carbon content inks printed with substantially lower ink film thicknesses. While this is partially due to the lower solid content, this also appears to be strongly influenced by rheology. The rheology of the inks also affected the quality of the printed lines, with lower viscosity inks being better able to slump and produce a more consistent line.

The composition of the ink also determined line reproduction through the print run. Given the large surface area and highly absorptive properties of carbon black in particular, there is a tendency for carbon inks to produce gradually thinner lines with more defects as the print run progresses; this tends to limit carbon ink to relatively large features. A lower carbon content in the ink will reduce drying in and might offer benefits in reproducing fine features and extending print runs. This will have to be weighed against the reduced conductivity but can be compensated for by printing thicker layers.

This study has demonstrated that the conductivity of a screen printed ink can be tuned, by modifying the ratios of graphite, carbon black and polymer to one another in the ink, depending on the application and performance required, but that this must be considered alongside the effect on print quality. A further consideration is the qualities of the component materials. Alternative source materials will have different electrochemical characteristics and particle sizes and will interact differently.
